# A systematic review on clinical management of antipsychotic-induced sexual dysfunction in schizophrenia

**DOI:** 10.1590/S1516-31802006000500012

**Published:** 2006-09-07

**Authors:** Anna Maria Niccolai Costa, Mauricio Silva de Lima, Jair de Jesus Mari

**Keywords:** Schizophrenia, Antipsychotic agents, Sexual and gender disorders, Libido, Esquizofrenia, Agentes antipsicóticos, Transtornos sexuais e da identidade sexual, Libido

## Abstract

**INTRODUCTION:**

Sexual dysfunction frequently occurs in patients with schizophrenia under antipsychotic therapy, and the presence of sexual side effects may affect compliance. The aim of this study was to review and describe clinical findings relating to the appropriate management of such dysfunctions.

**MATERIAL AND METHODS:**

The research was carried out through Medline (from 1966 to March 2005), PsycInfo (from 1974 to March 2005), and Cochrane Library (from 1965 to March 2005) and included any kind of study, from case reports to randomized trials.

**RESULTS:**

The most common sexual dysfunctions found in the literature were libido decrease, difficulties in achieving and maintaining erection, ejaculatory dysfunction, orgasmic dysfunction, and menstrual irregularities. Thirteen papers were found: eight of them were open-label studies, four were descriptions of cases, and only one was a randomized clinical trial. All of them were short-term and had small sample sizes. The agents used were: bromocriptine, cabergoline, cyproheptadine, amantadine, shakuyaku-kanzoto, sildenafil and selegiline.

**DISCUSSION:**

There was no evidence that those agents had proper efficacy in treating the antipsychotic-induced sexual dysfunction. An algorithm for managing sexual dysfunction induced by antipsychotics is suggested as a support for clinical decisions. Since the outcome from schizophrenia treatment is strongly related to compliance with the antipsychotics, prevention of sexual dysfunction is better than its treatment, since there is a scarcity of data available regarding the efficacy of intervention to deal with these problems.

## RESUMO

**INTRODUÇÃO:**

Disfunção sexual freqüentemente ocorre em pacientes com esquizofrenia em terapia com antipsicóticos e a presença de efeitos adversos sexuais pode afetar a adesão ao tratamento. O objetivo do estudo é rever e descrever achados clínicos relacionados ao manejo apropriado de tais disfunções.

**MATERIAIS E MÉTODOS:**

A pesquisa foi feita pelo Medline (de 1966 a março de 2005), PsycInfo (de 1974 a março de 2005) e Biblioteca Cochrane (de 1965 a março de 2005) e incluiu qualquer tipo de desenho de estudo de relato de caso a estudos clínicos randomizados.

**RESULTADOS:**

As disfunções sexuais mais comuns encontradas na literatura foram diminuição da libido, dificuldades em alcançar e manter ereção, disfunção ejaculatória, orgásmica e irregularidades menstruais. Treze artigos foram encontrados: oito deles eram estudos abertos, quatro descrições de casos e somente um estudo clínico randomizado. Todos eram de curta duração e com tamanho de amostra pequeno. Os agentes usados foram: bromocriptina, cabergolina, ciproheptadina, amantadina, shakuyaku-kanzo-to, sildenafil e selegilina.

**DISCUSSÃO:**

Não há evidências de eficácia apropriada destes agentes no tratamento da disfunção sexual induzida por antipsicóticos. Um algoritmo foi sugerido para manejo da disfunção sexual induzida por antipsicóticos, suportando decisões clínicas. Como o desfecho da esquizofrenia é fortemente relacionado a adesão ao tratamento com antipsicóticos, a prevenção da disfunção sexual é melhor que seu tratamento, visto que muito poucos dados estão disponíveis sobre a eficácia de intervenções destes problemas.

## INTRODUCTION

Schizophrenia treatment needs to cover several psychological and psychosocial interventions, and pharmacological treatment is essential for stabilizing the disease course and decreasing relapses. It is expected that virtually all patients with confirmed diagnoses of schizophrenia will receive antipsychotic drugs throughout their lives, and the efficacy of antipsychotic drugs has been confirmed in a number of randomized controlled trials. However, such drugs may induce some side effects such as acute Parkinsonism, tardive dyskinesia and neuroleptic malignant syndrome.^1-7^

Sexual dysfunction is often not specifically evaluated in clinical studies. When it affects patients on drug therapy, it affects their self-esteem, causes trouble for their sexual partners, interferes with their quality of life and compromises treatment compliance.^8^ Sexual dysfunction can be an important source of distress for patients and is one of the factors that must be taken into account when antipsychotic and anticholinergic drugs are selected to treat extrapyramidal symptoms.^9^ For example, in one study, a questionnaire was answered by 41 patients with schizophrenia under antipsychotic treatment, and it was detected that, among all the adverse effects and symptoms relating to mental disorders, the most important were the genital/sexual effects, and particularly impotence.^10^

Another publication involving a case-control study on the rates of sexual dysfunction among patients with schizophrenia in comparison with the general population indicated that male patients reported less desire for sex. These patients were less likely to achieve and maintain an erection, were more prone to premature ejaculation, and were less satisfied with the intensity of their orgasms. Female patients, however, reported less enjoyment.^11^

Although a high number of patients desire sexual relations (36.6% of men and 36.9% of women), sexual dysfunction is often observed in this population: 21.5% of men complain of erectile dysfunction and 18.7% of ejaculatory dysfunction; 19.3% of women and 15.9% of men complain of orgasmic dysfunction.^12^ Besides, 21.7% of women experience gonadal dysfunction as amenorrhea.^12^

The aim of this study is to review and to describe clinical findings related to the appropriate management of such dysfunctions.

## MATERIAL AND METHODS

A protocol for a systematic review was established in order to review the clinical management of antipsychotic-induced sexual dysfunctions in schizophrenia because of the importance of this subject in psychiatric clinical practice and its implications. The review was performed by seeking and selecting any studies related to this topic, from case reports to randomized clinical trials. The studies were located via electronic data sources, published citations and letters to authors. Reference searching was also added to the review.

Searches were performed in the following electronic databases: Medline (from 1966 to March 2005), PsycInfo (from 1974 to March 2005) and Cochrane Library (from 1965 to March 2005). For each database, studies were obtained and examined. The databases were searched using the following words: #1 - sexual OR erectile OR ejaculatory OR impotence OR menstrual OR hyperprolactinemia OR prolactin OR galactorrhea OR amenorrhea OR endocrine OR endocrinol*; #2 - dysfunct*; #3 - #1 and #2; #4 - antipsychotic* OR neuroleptic* OR dopamine; #5 - schizophren*; #6 - #4 and #5; and #7 - #3 and #6. The * symbol means that the beginning of the word was used (root term), allowing for the identification of more related terms.

The participants were individuals diagnosed with schizophrenia by any method of diagnosis. Those with schizoaffective disorder, schizophreniform disorder or psychotic illness were also included. All subjects were under treatment using either first or second antipsychotics. The study design was not taken into consideration since such a small number of studies were found. The clinical outcomes were those reported in the original studies on antipsychotic-related sexual dysfunction, and they included: erectile dysfunction, frigidity, anorgasmy, delayed ejaculation and other characteristics described in the studies.

## RESULTS

The search resulted in 13 papers: eight were open-label studies, four were descriptions of cases and only one was a randomized clinical trial. All of them were short-term and had small sample sizes, as shown in Table 1.^13-25^ Bromocriptine was involved in the treatment of these dysfunctions in two studies, sildenafil in four, amantadine in two and cyproheptadine, imipramine, shakuyaku-kanzo-to, cabergoline and selegiline in one each.

All of the antipsychotics that induced sexual dysfunction and which are described in Table 1 are first-generation antipsychotics, except for risperidone and olanzapine.

Cyproheptadine, a 5HT_2_ antagonist with antihistaminergic and adrenolytic properties, has also been used to improve sexual function and anorgasmy caused by antidepressants when taken in doses of 4 mg four times per day.^26^ As previously mentioned, there is one report in the literature on the use of imipramine in low doses (25-50 mg per day) for thioridazine-induced orgasmic disorder; however, the mechanism of action is not clear.^27^

Amantadine causes dopamine release at neuronal terminals. In patients with schizophrenia, amantadine decreases prolactin levels secondary to treatment with an antipsychotic. Amantadine also seems to improve sexual function when taken in doses of 100 mg per day in male patients.^19^ Bromocriptine, a dopamine agonist when administered in doses of 2.5 mg two or three times per day, may improve the libido of patients with hyperprolactinemia, normalize the menstrual cycle in amenorrheic patients and increase serum testosterone levels.^14^ However, it can also exacerbate psychosis. Another dopamine agonist is cabergoline, at a dose of 0.5 mg twice a week. Dopamine agonists such as bromocriptine and cabergoline may be successful in reducing the level of hyperprolactinemia and alleviating symptoms in some patients.^15^

Previous studies^14^ have shown that modest doses of bromocriptine may be safely prescribed to patients taking older first-generation agents, and who have symptomatic hyperprolactinemia without exacerbating psychosis.

Only three case reports and one openlabel trial mentioned the use of sildenafil in antipsychotic-induced dysfunction.^20-23^ These pharmacological strategies described above, once initiated, should not be discontinued until a minimum of two weeks of therapy have been administered.

Shakuyaku-kanzo-to (TJ-68) is a Japanese medicine that is composed of two herbs (*Radix paeoniae* and *Radix glycyrrhizae*) and has been used to treat acute muscle cramps including menstrual pains. TJ-68 has been investigated in relation to neuroleptic-induced hyper prolactinemia. Although the mechanism is unknown, TJ-68 may have a direct inhibitory effect on prolactin release from the pituitary. Alternatively, the observed effect of TJ-68 may be explained by an indirect action when reducing estradiol.^24,28^

Selegiline is a selective monoamine oxidase-B inhibitor; in low doses it selectively inhibits the oxidation of dopamine and phenyl- ethylamine.^29^ Its metabolites, L-amphetamine and L-methamphetamine, have sexual arousal properties.^25^ But in the only double-blind placebo-controlled trial found in the literature, selegiline did not show any effectiveness in improving sexual functioning, despite a significant decrease in prolactin levels.^30^

A description of the main drugs, respective doses, potential drug interactions and side effects can be found in Table 2.^13-20,23,24,27,30-38^

## DISCUSSION

Sexual performance among patients with schizophrenia may differ from the qualitative and quantitative patterns in the normal population. It may be altered through three main factors: by the disease itself as a consequence of affective and/or cognitive impairment; by antipsychotic drugs; and by other clinical problems (such as diabetes, hypertension, alcohol and drug abuse).^39,40-^ ^43^

In order to eliminate non-pharmacological factors, it is important to identify the factors that originated the sexual dysfunction, through evaluation of the patient's medical history and also physical and laboratory examinations. Both men and women should be asked specific questions regarding sexual dysfunction, and menstrual abnormalities should be evaluated during routine clinical evaluation. Furthermore, questions about dysfunction during autoerotic sexual activity (masturbation) and sexual intercourse are recommended in order to verify whether the dysfunction is provoked by the medication or by psychogenic causes.

After identifying the nature of the sexual dysfunction and its cause, it is important to evaluate the degree of patients' dissatisfaction and discomfort. The most common sexual disturbance is ejaculatory dysfunction induced by first-generation antipsychotics.^44^ Erectile dysfunction in males^45^ and loss of libido in both sexes have been documented among patients who use first-generation antipsychotics,^46^ including haloperidol, as well as among those who use second-generation antipsychotics (clozapine^47^ and risperidone^48^). Gonadal dysfunctions such as amenorrhea and galactorrhea can be found in these patients as well, and the estimated prevalence of amenorrhea in patients who use first-generation antipsychotics is from 15% to 50%,^49^ and 19% for galactorrhea.^50^ First-generation antipsychotics seem to create an increase in dose-dependent prolactin secretion^51,52^ and this effect suggests that is derived primarily from the pituitary.^53^

According to evidence relating to the use of serotonergic antidepressants, their influence on sexual function appear to be associated with decreased libido, erectile dysfunction and orgasmic dysfunction.^54^ Thus, the most likely explanation is that 5-HT2 receptor antagonism caused by second-generation antipsychotics may somehow contribute to the effect of prolactin elevation. These findings suggest that the specificity of second-generation antipsychotics, while presenting less dopamine receptor blockade in the tubularinfundibular system, may also exert a minor impact on prolactin levels.^55^

There are data suggesting that the incidence of sexual dysfunction differs according to the antipsychotic used. Second-generation antipsychotics have not been extensively studied with regard to sexual dysfunction, but they certainly differ from first-generation antipsychotics^56^ and also differ from each other.^57-61^ Several studies have shown an incidence of sexual dysfunction of 25-60% among patients treated with first-generation antipsychotics^45,62,63^ or with risperidone.^57-59,64^ 24% of the patients treated with clozapine and 45% of the patients treated with first-generation antipsychotics present with one or more sexual side effects, including erectile and ejaculatory dysfunction.^65^ Studies have shown different results: Knegtering et al.^57^ found olanzapine-induced sexual dysfunction in 27% of their schizophrenic patients; Montejo et al.^59^ found it in 10-33%; and Bobes et al. found it in 35.3% of patients. Quetiapine induced a lower rate of sexual dysfunction (18.2%), and risperidone the highest rate (43.2%).^60^ Thus, the findings in the literature indicate that all second-generation antipsychotics except for risperidone induce lower rates of sexual dysfunction.^66^ However, most of the sexual function evaluation data was not obtained from longitudinal studies, and base data, treatment duration, disease onset and illness severity were not taken into consideration. More studies are needed in order to have a better and clearer understanding.

**Table 1:** Description of published articles relating to sexual orgonadal dysfunction and use of antipsychotics

Dysfunction	Induced by	Number of patients and gender	Basic pathology	Therapy and dose	Study design	Results
Erection, ejaculation, libido (the paper also refers to amenorrhea, galactorrhea, weight change)	Fluphenazine, flupenthixol, pipotiazine, levomepromazine, cyamemazine, sulpiride	20 female 10 male	Mainly schizophrenia and some other psychiatric disorders	Bromocriptine 5-10 mg/day	Open-label non-controlled drug study^13^	Decreased serum prolactin level, weight loss, Return of menstrual cycle in 55%, relief of galactorrhea in 33%, improvement of erectile and ejaculatory dysfunction was less pronounced
Erectile (the paper also refers to amenorrhea, galactorrhea)	Probably only first-generation antipsychotics, on the basis of the date of the study	24 female 11 male	Schizophrenia and other psychiatric disorders	Bromocriptine 5-7.5 mg/day	Open-label non-controlled drug study^14^	Return of menstrual cycle in 70%, relief of galactorrhea in 80% and improvement of impotence in 66%
Hypogonadism (the paper also refers to hyperprolactinemia)	Risperidone	4 female 1 male	Psychotic disorders	Cabergoline 0.5 mg twice a week or bromocriptine 7.5 mg/d to 25 mg/d	Case series^15^	In 3 out of 4 patients such additional therapy reduced the prolactin level and alleviated hypogonadism. None of the patients treated with these agents had worsening of psychosis
Ejaculatory	Flupenthixol	1 male	Schizophrenia	Cyproheptadine 8 mg/day	Case report^16^	Returned to satisfactory ejaculation
Orgasmic	Thioridazine	8 male	Schizophrenia	Imipramine 25-50 mg/day	Open-label non-controlled drug study^17^	50% returned to previous ejaculatory function
Prolactin-mediated neuroendocrine side effects	Neuroleptic	4 female 6 male	Schizophrenia	Amantadine 200-300 mg/day	Open-label reversal drug study^18^	Significant reduction in all neuroendocrine side effects: serum prolactin levels, body weight, gynecomastia/galactorrhea, breast tenderness, decreased libido and amenorrhea
Desire, erection, ejaculation, satisfaction with sexual performance	First-generation antipsychotics (haloperidol, thioridazine, fluphenazine, propericiazine	12 male	Schizophrenia	Amantadine 100 mg/day	Open-label non-controlled drug study^19^	Improvement in all events evaluated except ejaculation. Decreased serum prolactin
Decreased libido and erection	Sulpiride Risperidone	1 male	Schizophrenia	Sildenafil 50 mg/week 50 mg every 3 weeks	Case report^20^	Improvement in sexual performance
Erectile	Haloperidol	1 male	Schizoaffective	Sildenafil 50 mg	Case report^21^	Obtained complete erection and satisfactory sexual intercourse
Erectile	Olanzapine	10 male	Not mentioned	Sildenafil 50-100 mg	Open-label non-controlled drug study^22^	40% of the patients were considered to be "very much improved" and 30% "much improved". 80% were receiving 50 mg
Erectile	Risperidone	12 male	Schizophrenia	Sildenafil starting dose 25 mg with the possibility of increasing the dose to 75 mg	Open-label non-controlled drug study^23^	67% of the patients exhibited partial or great improvement
Hyperprolactinemia Sexual dysfunction	Neuroleptic — not mentioned which one	20 male	Schizophrenia	Shakuyaku-kanzo- to (TJ-68) 7.5 g (2.5 g three times daily, orally) for 4 weeks	Open-label non-controlled drug study^24^	In five patients, prolactin levels decreased by more than 50%. Three of the ten patients who had complained of reduced sexual desire, experienced subjective improvement
Hyperprolactinemia Sexual dysfunction	Perphenazine or haloperidol	10 male	Schizophrenia	Selegiline 15 mg/day for 3 weeks	Randomized, double-blind, placebo-controlled crossover study^25^	Not effective in improving any domain of sexual functioning despite a significant decrease in prolactin levels

**Table 2:** Main drugs and respective dosages used in treating antipsychotic-induced sexual dysfunction

Drug	Dose	Mechanism of action for sexual dysfunction	Side effects^31^	Potential psychiatric drug interactions^31^
Bromocriptine	2.5 mg 2-3 x/d	Dopaminergic agonist13,14,32-35	Constipation (3-14%), nausea (50%), vomiting, dizziness (17%), fatigue (7%), headache (19%), hypotension, peripheral vasoconstriction, cerebral ischemia, seizures (rare), stroke (rare), confusion, dyskinesia, hallucinations, psychosis, pleuropulmonary changes (long-term use), postpartum myocardial infarction (rare)	May induce or exacerbate psychosis in psychiatric patients, but this occurs primarily in those with a psychotic diathesis and who are not currently receiving neuroleptic medication. Factors to be considered: dose of bromocriptine, duration of treatment, and the clinical state of the patient
Cabergoline	0.5 mg twice a week	Dopaminergic agonist^15,36^	Dizziness, fatigue, headache, constipation, nausea, somnolence, depression, orthostatic hypotension (1-10%), pleural effusion (rare), pulmonary fibrosis (rare), abdominal pain (4-5%), vertigo (4-5%)	May decrease therapeutic effect of antipsychotics
Cyproheptadine	4 mg 4 x/d	Serotonergic antagonist (5-HT2)^16^	Central nervous system depression, dry mouth, drowsiness, increased appetite, weight gain, nausea, vomiting, diarrhea, abdominal discomfort, thickening of bronchial secretions, hepatitis	Decreased fluoxetine efficacy, prolonged and intensified anticholinergic effects when used with MAOI, reduced paroxetine efficacy
Amantadine	100-300 mg/d	Increases dopamine release and reduces dopamine and noradrenaline reuptake into synaptic terminals^18,19,27^	Dizziness, insomnia (most common), agitation, anxiety, confusion, depression, dream abnormality, fatigue, hallucinations, headache, irritability, nervousness, nausea (most common), anorexia, constipation, diarrhea, dry mouth, orthostatic hypotension, peripheral edema, neuroleptic malignant syndrome, exacerbation of mental problems, suicide attempts	Bupropion: increased risk of adverse effects; zotepine: decreased pharmacological effect of amantadine, may decrease therapeutic effect of antipsychotics
Shakuyaku-kanzo-to (TJ-68)	2.5 g three times daily	The mechanism is unknown: either a direct inhibitory effect on prolactin release from the pituitary or an indirect action via a reduction in estradiol, or both^24,28^	Nausea, pseudo-hyperaldosteronism,^37^ myoglobinuria (rare); long term effect: may decrease serum testosterone levels,^38^ use is not recommended for pregnant women or for people with liver and kidney disorders^37^	No psychiatric drug interactions known Thiazide diuretics: increased potassium loss; digitalis: more sensitivity to digitalis due to hypokalemia
Sildenafil	50 mg/week	Phosphodiesterase inhibitor^20-23^	Flushing (4-10%), dizziness (2%), headache (11-16%), diarrhea (4%), dyspepsia (4-8%), abnormal vision, nasal congestion, skin rash (2%), myocardial infarction (rare), priapism (rare)	No psychiatric drug interactions known Nitrates: severe hypotensive effect; clarithromycin, erythromycin: increased risk of sildenafil effects
Imipramine	25-50 mg/d	Unknown^17^	Blurred vision, drowsiness, dizziness, weakness, fatigue, headache, dry mouth, constipation, bloating, urinary retention, weight gain, agranulocytosis (rare), arrhythmia, atrioventricular conduction changes, heart block, palpitations, jaundice and hepatic dysfunction (rare), orthostatic hypotension, syncope, hypertension, psychotic reactions (rare), seizures (rare)	Antipsychotic: increased risk of cardiotoxicity; barbiturates: possible decreased TCA serum concentrations and possible additive adverse effects; carbamazepine: decreased imipramine effectiveness; citalopram: increased in the bio-availability and half-life of desipramine; fenfluramine: increased risk of imipramine (sedation); phenothiazine: increased levels and toxicity with either agent; fluvoxamine: increased imipramine levels and signs of toxicity; MAOI: neurotoxicity, seizures or serotonin syndrome; paroxetine: imipramine toxicity; phenytoin: increased risk of phenytoin toxicity; sertraline: modest elevations in imipramine serum levels; St John's wort: increased risk of serotonin syndrome; tobacco: decreased imipramine concentrations; ziprasidone: increased risk of cardiotoxicity; zolmitriptan: increased risk of cardiotoxicity
Selegiline	15 mg/d	Selective monoamine oxidase B inhibitor^30^	Abdominal pain, nausea, dizziness, lightheadedness, confusion, hallucinations	TCA: neurotoxicity, seizures, or serotonin syndrome; bupropion: increased bupropion toxicity; carbamazepine: hypertensive urgency, hyperpyrexia, and seizures; citalopram: central nervous system (CNS) toxicity or serotonin syndrome; buspirone, phenelzine, phentermine, amphetamine, and amphetamine-like: hypertensive crisis; antipsychotic: increased risk of cardiotoxicity; fluoxetine, fluvoxamine, sibutramine, sertraline, and paroxetine: CNS toxicity or serotonin syndrome; reboxetine and nefazodone: hyperthermia, rigidity, myoclonus, seizures, fluctuations of vital signs, or mental status changes; phenobarbital: CNS sedation; St John's wort: increased risk of serotonin syndrome and/or increased risk of hypertensive crisis; triptans: serotonin syndrome; tramadol: nausea, vomiting, cardiovascular collapse, respiratory depression, seizures

MAOI = monoaminooxidase inhibitor; TCA = tryciclic antidepressant.

**Figure 1:**
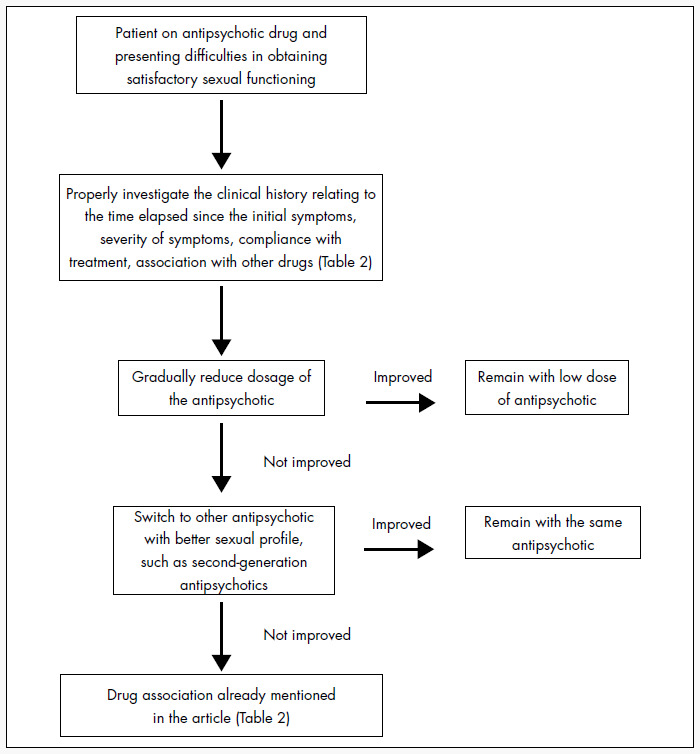
Clinical management of antipsychotic-induced sexual dysfunction in schizophrenia.

## CONCLUSION AND RECOMMENDATIONS

Kaplan^67^ observed that several patients suffering from schizophrenia who presented with sexual dysfunction had sought sexual therapy prior to the onset of the psychotic phase of the disease. The author mentioned that sexual therapy should only be administered once it is certain that the illness is in a remission phase and that the sexual dysfunction is not drug-induced or a defense against a possible episode of illness. This author also expressed concern regarding the use of sexual therapy among subjects with sexual dysfunction, since the resolution of these symptoms may interrupt the homeostasis of the patient and ultimately cause a relapse. This risk should be evaluated on behalf of such patients and respective partners, and they should be provided with the necessary information, be advised to practice satisfactory sexual experiences and be warned against inappropriate behavior.

The aim of a systematic review is to thoroughly assess the best possible evidence about the effects of a healthcare intervention or treatment in a particular healthcare situation, by means of a set procedure. However, in this case, due to the lack of double-blind studies and the bias of non-publication of negative treatments and strategies, it is impossible to estimate the relative efficacy of the interventions available for treating antipsychotic-induced sexual dysfunction. In order to avoid iatrogenic sexual dysfunction in schizophrenia, patients should first be given the option of an appropriate drug, as discussed above. The patients who present higher risk of sexual disorders are those receiving first-generation antipsychotics and, more specifically, those taking high doses of these drugs (dose-dependent effect).^68^ In cases where a drug-induced sexual dysfunction is present, it is advisable to reduce first-generation antipsychotics to lower doses that are still effective, maintaining the clinical status. Moreover, if the patient can be maintained on a low dose, the incidence of other adverse symptoms that may interfere in sexual activity, such as sedation and Parkinsonism, will be reduced.

Withdrawal of a first-generation antipsychotic, or switching from a first-generation to a prolactin-sparing second-generation antipsychotic, should be done if hyperprolactinemia is present. Changes in antipsychotic input may also alter sexual function. If the dysfunction persists while the patient is receiving anticholinergics, the drug should be discontinued since there is evidence that anticholinergic properties found in some antidepressants can cause sexual dysfunction.^69^ Acetylcholine will probably not play a direct role in sexual function; however, it may be important in the adrenergic-cholinergic balance that is necessary for normal sexual function.^70^

Although the more favorable benefit-versus-risk ratio of the new antipsychotics represents a major improvement over the older neuroleptics, differences need to be addressed and more clearly documented. There was an almost complete lack of randomized controlled trials (RCTs) on the clinical management of antipsychotic-induced sexual dysfunction in the literature, and no proper comparisons of the efficacy of agents. Further clinical trials focusing on the sexual sphere are necessary in order to progress with regard to compliance and distress among patients with schizophrenia.

On the basis of the scarce data available, an attempt was made to design a preliminary algorithm for the clinical management of antipsychotic-induced sexual dysfunction, as described in Figure 1. It is important to point out that such an algorithm should be analyzed within the context of the patient's overall clinical condition, avoiding radical interventions that could threaten the patient's clinical stability.

In conclusion, the findings in the literature indicate that antipsychotics may have different profiles of adverse sexual effects, but the results are inconclusive since most of the sexual function evaluation was not performed in longitudinal studies. Moreover, base data, treatment duration, disease onset and illness severity were not taken into consideration.

Adverse sexual effects must be diagnosed, and should be discussed clearly and treated while maintaining the patient's mental status, treatment compliance and quality of life. More studies are needed in order to provide physicians with better understanding of this problem, thereby leading towards efficacious and safe solutions.
